# Sero-prevalence and associated risk factors for hepatitis C virus infection among voluntary counseling testing and anti retroviral treatment clinic attendants in Adwa hospital, northern Ethiopia

**DOI:** 10.1186/s13104-016-1936-3

**Published:** 2016-02-23

**Authors:** Ataklti Hailu Atsbaha, Tsehaye Asmelash Dejen, Rashmi Belodu, Konjit Getachew, Muthupandian Saravanan, Araya Gebreyesus Wasihun

**Affiliations:** Department of Medical Microbiology and Immunology, Institute of Biomedical Science, College of Health Sciences, Mekelle University, P.O.Box: 1871, Mekelle, Ethiopia; Tigray Regional Health and Research Laboratory, P.O.Box: 1807, Mekelle, Ethiopia

**Keywords:** HCV, Sero-prevalence, Risk factors, VCT clinic, ART clinic, Adwa hospital

## Abstract

**Background:**

Hepatitis C virus (HCV) is a major health concern where about 3 % of the world’s population is infected globally. In Ethiopia the prevalence ranges from 0.9 to 1.3 % in the general populations. Human immune deficiency virus (HIV) patients due to their weak immune response are heavily affected by the virus. There is no data on magnitude and associated risk factors for HCV infection among voluntary counseling, testing center and anti retroviral treatment clinic Attendants in the study area. Therefore, the aim of this study was to determine the sero-prevalence and associated risk factors for HCV infection among voluntary counseling testing and anti retroviral treatment clinic attendants Adwa general hospital.

**Methods:**

Cross sectional study was carried out among 302 participants (151 HIV-negative from VCT and 151 HIV-positive from ART follow up) clinics of Adwa hospital from September to December, 2014. About 5 ml of venous blood samples were collected from study participants for anti HCV antibody tests. Univariate analyses were used to identify associated variables with anti HCV positivity. Variables having p < 0.05 were considered as statistically significant association.

**Results:**

Out of the total 302 participants, 52.6 % of them were females and 47.4 % males. The mean age of the participants was 34.1 year (SD ± 10.5). The overall sero-prevalence of HCV in this study was 4.3 %. The prevalence HCV (6.6 %) was higher among the ART clinic attendants than the VCT (2 %) clinic attendants. History of hospitalization (p = 0.001), tooth extraction (p = 0.018) and blood transfusion (p = 0.041) showed statistically significant association with anti-HCV antibody.

**Conclusion:**

HCV sero-prevalence in this study was high. The prevalence was three fold higher among HIV positive patients than their counter parts. Thus, screening of HCV should be done among HIV patients for close monitoring and better management in HIV patients.

## Background

Infection with hepatitis C virus (HCV) is a major cause of chronic hepatitis, cirrhosis, and hepato cellular carcinoma around the world [1]. About 3 % of the world’s population have HCV with 170 million chronic carriers at risk of developing liver cirrhosis, and annual new infection rate of four million [2]. The prevalence of HCV is higher in developing nation like the sub-Saharan Africa [3].

Above 80 % of the HCV exposed individuals become chronically carriers, and 30 % of them develop chronic liver disease [4]. High-risk groups who have exposure with blood or blood products, like intravenous drug use, patients with pediatric hematologic malignancies, long term haemo-dialysis, with organ transplantation, infants born to HCV positive mothers and un-protective sex are highly infected with HCV [5].

Due to the similar routes of transmissions and exposure, HIV patients are highly infected with HCV; as a result there are about four to five million HIV/HCV co-infection globally [4]. As HIV virus declines the immune response of the patients to HCV, this results in lower viral clearance of HCV infection, increased levels of HCV RNA in the blood, rapid progression to HCV-related end stage liver disease and increased risk of antiretroviral associated liver toxicity [6]. These HCV related complication in HIV patients highlights the importance of timely and appropriate diagnosis and treatment to prevent further spread of HCV infections and improve their quality of life [7].

Hence, magnitude of HCV infections in patient populations in different areas and risk factors for the transmission should be investigated to take measures for reduction of such transmission in the populations. In Ethiopia there are few studies done on prevalence of hepatitis infections [8–15] and HCV/HIV co-infection [16–19].

Although screening based on antibody detection could markedly reduce the risk of HCV infection [20], little emphasis is given for viral hepatitis co-infections in HIV patients in Ethiopia and recent ART guidelines don’t recommend routine screening tests for HCV [21], rather recommends only for alanine amino transferase (ALT) levels test to determine liver related complications among HIV patients. The magnitude of HCV among HIV positive and negative participants and its associated risk factors isn’t known in the study area. Therefore this study was aimed to fill the existing knowledge gap on HCV prevalence in the study area.

## Methods

### Study design, study period and sample size determination

Prospective cross**-**sectional study was conducted from September to December, 2014 at Adwa hospital which is located in Central Zone of Tigray regional state, northern Ethiopia. Three hundred two study participants (151 HIV-negative from VCT and 151 HIV-positive subjects in ART follow up) were enrolled in the study. Adwa town is located at longitude and latitude of 14°15′N and 38°54′E at an elevation of 1907 meters above sea level. It is 1006 km north of Addis Ababa (the capital city of Ethiopia) and 223 km far from North–West of Mekelle, the capital city of Tigray national regional state. Adwa General hospital covers four catchment Woredas and serves for an estimated population of more than 970,644. Adwa general hospital is the main center for ART and VCT center in the zone.

### Sample size determination

The sample size was calculated using a double proportion formula by considering the seroprevalence of HCV in HIV positive (p1) and HIV negative study participants (p2) which is 9.2 and 1.58 %, respectively Hadush et al. 2013 [18]. Using the following formula:

$${\text{n}}1 = {\text{n}}2 = \frac{{\left( {{\text{Z}}\upalpha/2 \sqrt {\left( {1 + \frac{1}{\text{r}}} \right)} \overline{\text{pq}} + {\text{Z}}\beta \sqrt {{\text{p}}1{\text{q}}1 + \frac{{{\text{p}}2{\text{q}}2}}{\text{r}}} } \right)^{2} }}{{\left( {{\text{p}}1 - {\text{p}}2} \right)^{2} }}$$

${\bar{\text{p}}} = \frac{{{\text{p}}1 + {\text{p}}2}}{2}$ average proportion, ${\bar{\text{q}}} = 1 - {\bar{\text{p}}}$r = Ratio of HIV positive to HIV negative individuals = n1/n2 = 1 for equal sample size.

P1 = Prevalence of HCV among HIV positive individuals.

P2 = Prevalence of HCV among HIV negative individuals.

${\text{Z}}\upalpha/2$ = The z**-**score corresponding to the probability with which it is desirable to be able to conclude that an observed difference of size (p1 − p2) of variables between HIV positive and HIV negative individuals will not occur by chance = 1.96.

Zβ = the score corresponding to the degree of confidence with which it is desired to certain of detecting a difference size (p1 − p2) between variables of that actively present = 0.84.

$${\bar{\text{p}}} = \frac{{{\text{p}}1 + {\text{p}}2}}{2} = \frac{0.092 + 0.0158}{2} = 0.0539$$

$${\bar{\text{q}}} = 1 - {\bar{\text{p}}} = 1 - 0.0539 = 0.9461$$

p1 = 0.092 and q1 = 0.908

p2 = 0.0158 and q2 = 9842

$${\text{n}}1 = {\text{n}}2 = \frac{{\left( {1.96\sqrt {1 + \frac{1}{1}} \left( {0.0539 \times 0.9461} \right) + 0.84\sqrt {\left( {0.092 \times 0.908} \right) + \left( {\frac{0.0158 \times 0.9842}{1}} \right)} } \right)^{2} }}{{\left( {0.092 - 0.0158} \right)^{2} }}$$

${\text{n}}1 = {\text{n}}2 = \frac{0.79273}{0.005806} = 137$ from each group with a total of 274 participants. However, to increase the power of the study we added 10 % contingency, $137 \, \times \frac{10}{100} = \sim14$, and 14 + 137 = 151 in each group with a total of 302 participants.

### Inclusion and exclusion criteria

All HAART naive adult HIV positive study participants who visited ART clinic for their pre ART follow up and other laboratory investigation during the study period were included in the study. Individuals who visited VCT clinic and sero negative for HIV testing regardless of other risk factors during the study period were included in the study. Children, and individuals who refused and unable to give informed consent were excluded from the study.

### Data collection procedure

Socio-demographic data and risk factors such as, drug injection, dental procedure, surgery, blood transfusion, hospitalization, history of tattooing, scarification, multiple sexual partners, abortion, STI and visiting traditional healers were collected using standard questioner by clinical nurses working in the hospital.

### Blood sample collection and processing

Approximately 5 ml of venous blood was collected using vacationer tube and then serum was separated by centrifugation at 3000 rpm for 10 min. Clear non-hemolysed serum specimens were tested for HCV by Anti-HCV Rapid Test kits (Zhejiang Orient Gene Biotech Co.LTD, China) according to the manufacturer’s instruction. The presence of antibodies to HIV for the VCT attendants was determined using the standard methods available to determine the test in the hospital.

### Quality control and data analysis

The Quality of the study was maintained by strictly following standard operational procedures during laboratory investigation. Internal quality control was done using known anti-HCV-antibody positive and anti-HCV-antibody negative samples. The data was entered and analyzed using SPSS version 20.0 statistical software. Chi square test (χ^2^) was used to see the association with HCV and *p* value of less than 0.05 was considered as statistically significant.

### Ethical consideration

Ethical clearance was obtained from the Ethical Review committee of the College of Health Science, Mekelle University (Ref. no: ERC 0459/2014). Official letter of permission was obtained from Tigray Regional Health Bureau and Adwa General Hospital. Written consent was obtained from all study participants. Confidentiality was kept and participants who were found to be positive for HCV were communicated by the health care workers for further management.

## Results

### Socio-demographic and sero-prevalence of HCV among participants

Among the total 302 study participants of VCT and ART clinics (response rate 100 %), 52.6 % of them were females and 47.4 % were males. The mean age of participants was 34.1 years (SD ± 10.5). One hundred and nineteen (39.4 %) of the participants were in the age range of 18–29 years. Fifty percent of the study participants were single, and 47.0 % of them have attained secondary school or above.

The overall seroprevalence of anti**-**HCV antibody in this current study was 13 (4.3). HCV prevalence was relatively higher in the age group of 40–49 years and in male, but not statistically significant (p > 0.5). Similarly higher sero-prevalence was seen among divorced, farmers and illiterate participants but not statistically significant (Table 1). In this study, HCV infection was higher in HIV positive patients 10 (6.6 % than the negative 3 (2 %) patients (Table 2).

**Table 1:** Seroprevalence of HCV by socio-demographic variables among VCT and ART clinic attendants in Adwa general hospital from September to December, 2014

Variables	HCV-antibody			χ^2^	p value
	Positive no (%)	Negative no (%)	Total no (%)		
Age group					
18–29	4 (3.4)	115 (96.6)	119 (39.4)	0.975	0.807
30–39	4 (4.3)	58 (93.5)	62 (20.5)		
40–49	4 (6.5)	26 (96.3)	27 (8.9)		
≥50	1 (3.7)	90 (95.7)	119 (39.4)		
Sex					
Male	7 (4.9)	136 (95.1)	143 (47.4)	0.038	0.845
Female	6 (3.8)	153 (96.2)	159 (52.6)		
Marital status					
Married	3 (2.9)	99 (97.1)	102 (33.8)		
Single	7 (4.6)	145 (95.4)	152 (50.3)		
Divorced	2 (6.9)	27 (93.1)	29 (9.6)	1.009	0.799
Widowed	1 (5.0)	18 (94.7)	19 (6.3)		
Educational status					
Illiterate	4 (7.4)	50 (92.6)	54 (17.9)		
Elementary school	5 (4.7)	101 (95.3)	106 (35.1)	2.069	0.355
High school and above	4 (2.8)	138 (97.2)	142 (47.0)		
Occupational category					
Unemployed	4 (4.2)	91 (95.8)	95 (31.5)		
Private employee	5 (4.7)	102 (95.3)	107 (35.4)		
Government	1 (2.6)	38 (97.4)	39 (12.9)	0.382	0.996
Farmer	1 (5.0)	19 (95.0)	20 (6.6)		
House wife	1 (4.8)	20 (95.2)	21 (7.0)		
Sex worker	1 (5.0)	19 (95.0)	20 (6.6)

**Table 2:** Comparison of HIV/HCV coinfection between VCT and ART clinics at Adwa hospital (September to December, 2014.)

HIV status	HCV-antibody			χ^2^	p value
	Positive N (%)	Negative N (%)	Total no (%)		
Positive	10 (6.6)	141 (93.4)	151 (50)	4.146	0.085
Negative	3 (2.0)	148 (98.0)	151 (50)		
Total	13 (4.3)	289 (96.7)	302 (100)		

### Associated risk factors and HCV sero-prevalence

In this study, previous history of hospitalization (χ^2^ = 15.635, p = 0.001), tooth extraction (χ^2^ = 5.569, p = 0.018) and blood transfusion (χ^2^ = 4.349, p = 0.041) have shown a statistically significant association with sero-prevalence of HCV. Dental procedure, surgery, multiple sexual partners, history of abortion, STI and visiting traditional healers however; showed no statistically significant association with HCV infection (p > 0.05) (Table 3).

**Table 3:** Univariate analysis of HCV sero-prevalence by risk factors among VCT and ART clinic attendants in Adwa general hospital from September to December, 2014

Risk factors	HCV-antibody			χ^2^	p value
	Positive N (%)	Negative N (%)	Total N (%)		
Tattooing/scarification					
Yes	3 (10.0)	27 (90.0)	30 (9.9)	2.018	0.128
No	10 (3.7)	262 (96.3)	272 (90.1)		
Unsafe injection					
Yes	2 (12.5)	14 (87.5)	16 (5.3)	1.908	0.146
No	11 (3.8)	275 (96.2)	286 (94.7)		
Visiting traditional healers					
Yes	2 (7.7)	24 (92.3)	26 (8.6)	0.662	0.310
No	11 (4.0)	265 (96.0)	276 (91.4)		
Hospitalization					
Yes	10 (12.8)	68 (87.2)	78 (25.8)	15.635	0.001
No	3 (1.3)	221 (98.7)	224 (74.2)		
Tooth extraction					
Yes	4 (15.4)	22 (84.6)	26 (8.6)	5.569	0.018
No	9 (3.3)	267 (96.7)	276 (91.4)		
Surgical operation					
Yes	2 (8.7)	21 (91.3)	23 (7.6)	0.931	0.259
No	11 (3.9)	268 (96.1)	279 (92.4)		
Blood transfusion					
Yes	2 (25.0)	6 (75.0)	8 (2.6)	4.349	0.041
No	11 (3.7)	283 (96.3)	294 (97.4)		
Occupational exposure to blood					
Yes	1 (8.3)	11 (91.7)	12 (4.0)	0.395	0.416
No	12 (4.1)	278 (95.9)	290 (96.0)		
Multiple sexual partners					
Yes	4 (6.6)	57 (93.4)	61 (20.2)	0.850	0.305
No	9 (3.7)	232 (96.3)	241 (79.8)		
STI					
Yes	3 (9.4)	29 (90.6)	32 (10.6)	1.761	0.148
No	10 (3.7)	260 (96.3)	270 (89.4)		
Abortion					
Yes	2 (6.9)	27 (93.1)	29 (9.6)	0.815	0.301
No	4 (3.1)	126 (96.9)	130 (90.4)		

## Discussion

Sero-prevalence of HCV among HIV negative and HIV positive in this study was (4.3 %, with prevalence rate of 2 and 6.6 % in HIV negative and HIV positive participants respectively. The overall sero-prevalence of HCV in this study (4.3 %) was comparable with results from Burkina Faso 5.4 % [22], Malawi 4.5 % [23] and Ghana 3.6 % [24], however, it was higher than reports from Addis Ababa, 0.9 % [17] and Debretabor, South Gondar 1.3 % [12]. Higher value of prevalence than our result was reported from other parts of Ethiopia, Mekelle, 6 % [18] and South Gondar 7.5 % [8].

HCV/HIV coinfection rate in this study, 6.6 % was comparable with the studies done in Gonder, 5.0 % [25], Mekelle, 8.6 % [18], Nigeria, 8.2 % [26] and Malawi, 5.7 % [27]. Our result was however; higher than reports from other parts of Ethiopia like Debretabor Hospital, South Gondar, 1.3 % [12], Bahir Dar, 5.5 % [15], Northwest Ethiopia, 1.7 % [13], and Burkina Faso, 4.8 % [22], Malawi, 4.5 % [23], Zimbabwe, 0.8 % [28], Zambia, 2.2 % [29], Rwanda 4.9 % [30], Ghana, 3.6 % [24], SouthAfrica, 1 % [31], Gambia, 0.6 % [32], Zambia, 1.2 % [33], Senegal, 1.6 % [34], Uganda, 3.3 % [35] and Cote D’voir, 1.2 % [36].

Higher HCV/HIV coinfection was documented from Hawassa, South Ethiopia, 9.2 % [19] and Addis Ababa, 11.6 % [17], Cameroon, 8.6 % [37] and Kenya, 10.3 % [38].

These variations could be due to differences in geographic regions, types of risk exposure and methodology used [39, 40]. The higher HCV co-infection rate in HIV patients in this study and other places could be due to the shared modes of transmission of both viruses in the study patients [17, 41]. Hence investigation of HCV in HIV-positive patients is crucial to prevent them from further infections and complications [42].

HCV prevalence among VCT clinic attendants in this current study (2 %) was comparable with results from Mekelle, Ethiopia, 1.65 % [18]; but lower than that of Addis Ababa (5 %) [16].

There was no statistically significant association between sero-prevalence rate of HCV and marital status, educational status and occupational status of the participants (p > 0.05). This was in line with finding by of other researchers from Zambia [33] and Gonder [25].

However, hospitalization, tooth extraction and blood transfusion showed statistically significant association with HCV infection (p < 0.05). This was supported by other reports from Ethiopia [12, 18]. On the other hand, these variables were not significantly associated with HCV infection in the study conducted in Hawassa [19] and Addis Ababa [17]. None of the HCV antibody positive study participants had history of intravenous drug use. This might be due to the fact that it is uncommon practice in the study area.

### Limitation

Rapid test kit was used to detect anti HCV prevalence, hence may not detect early HCV infections like what the ELISA and PRC tests do.

## Conclusion

The overall sero prevalence of HCV in this study was high; hospitalization, tooth extraction and blood transfusion were significantly associated with HCV infection. The prevalence was three times higher among HIV positive than their counter parts. Therefore, routine screening programme of HCV infection in HIV-infected patients is should be in place for the better management of the patients and prevent them from further complications. Implementation of more effective public health education and counseling on the risk factors should be given to reduce the burden of HIV/HCV co-infection.
